# Estimating CO_2_/N_2_ Permselectivity through Si/Al = 5 Small-Pore Zeolites/PTMSP Mixed Matrix Membranes: Influence of Temperature and Topology

**DOI:** 10.3390/membranes8020032

**Published:** 2018-06-16

**Authors:** Clara Casado-Coterillo, Ana Fernández-Barquín, Susana Valencia, Ángel Irabien

**Affiliations:** 1 Department of Chemical and Biomolecular Engineering, Universidad de Cantabria, Av. Los Castros s/n, 39005 Santander, Spain; fbarquina@unican.es (A.F.-B.); irabienj@unican.es (Á.I.); 2 Instituto de Tecnología Química, Universitat Politècnica de València-Consejo Superior de Investigaciones Científicas, Av. de los Naranjos s/n, 46022 Valencia, Spain; svalenci@itq.upv.es

**Keywords:** mixed matrix membranes, Poly(trimethylsilyl-1-propyne) (PTMSP), small-pore zeolites (CHA, RHO, LTA), temperature, modeling

## Abstract

In the present work, the effect of zeolite type and topology on CO_2_ and N_2_ permeability using zeolites of different topology (CHA, RHO, and LTA) in the same Si/Al = 5, embedded in poly(trimethylsilyl-1-propyne) (PTMSP) is evaluated with temperature. Several models are compared on the prediction of CO_2_/N_2_ separation performance and then the modified Maxwell models are selected. The CO_2_ and N_2_ permeabilities through these membranes are predicted with an average absolute relative error (AARE) lower than 0.6% taking into account the temperature and zeolite loading and topology on non-idealities such as membrane rigidification, zeolite–polymer compatibility and sieve pore blockage. The evolution of this structure–performance relationship with temperature has also been predicted.

## 1. Introduction

Carbon capture strategies are still envisaged as one of the major challenges for preventing CO_2_ emissions to the atmosphere from anthropogenic sources. Membrane separation technology is often presented as an energy efficient and economical alternative to conventional capture technologies although not yet passing through the stage of pilot plant scale [1]. Polymer membranes for CO_2_ separation are especially constrained by a performance ‘upper bound’ trade-off between gas permeability and selectivity, which becomes especially significant for treating large volumes of flue gas. The simultaneous improvement on membrane permeability and selectivity is very attractive for industrial applications. Mixed matrix membranes (MMMs), which consist of the introduction of small amounts, usually below 30 wt %, of a special filler providing properties such as a molecular sieve, ion-exchange and robustness in a processable polymer matrix [2], are surpassing this upper bound [3,4,5,6,7]. More than homogenous distribution, the main challenge of MMM fabrication is achieving a good adhesion and compatibility between the inorganic filler and the polymer, avoiding the voids and defects that deteriorate separation performance [8].

Polyimide materials have been, firstly, studied for gas separation because of their stability and selectivity. However, permeability is usually low for CO_2_ separation [9]. The first and most widely used fillers are zeolites since the pioneering work of Zimmermann et al. [10]. Recently, zeolite 5A was introduced in Matrimid to prepare MMMs for CO_2_/CH_4_ separation, after particle surface modification to obtain a defect-free membrane [11]. Amooghin et al. [12] reported the ion exchange effect of Ag^+^ in zeolite Y-filled Matrimid MMMs led to a CO_2_ permeability increase of 123% from 8.64 Barrer in pure Matrimid to 18 Barrer in 15% AgY-filled MMM, where 1 Barrer is defined as 10^−10^ cm^3^(STP) cm cm^−2^ s^−1^ cmHg^−1^.

A simple approach to produce high permeability and selectivity membranes without the use of modifiers that complicate the synthesis procedures is the variation of the inorganic particles composition themselves to influence the polarity in comparison with the selected polymer matrix. In the case of zeolites, this is represented by the Si/Al ratio and determines many properties of the material, including ion exchange capacity [13]. Thus, for the development of high perm-selective membrane materials for CO_2_ separation, we focused on the most permeable polymer, poly(trimethylsilyl-1-propyne), PTMSP, and observed that the adhesion with LTA fillers and therefore CO_2_/N_2_ separation properties were best with a low Si/Al ratio even upon increasing temperature [14]. The strong influence of zeolite topology on CO_2_ adsorption has also been acknowledged [15], giving the possibility to locally tune the energy interactions, promoting size and shape selectivity and clustering. However, this effect is not always straightforward because most zeolites cannot be synthesized in pure silica form or at similar Si/Al compositions. Exceptions to this rule are LTA (ITQ-29) [16] and CHA [17]. To avoid this and to see that the lower Si/Al favored the compatibility with glassy hydrophobic PTMSP [14], we fixed an intermediate value of the Si/Al ratio to 5, in order to study the influence of the zeolite filler topology using different small pore zeolites (LTA, CHA, RHO) in the CO_2_/N_2_ separation of PTMSP-based MMMs in the temperature range 298–333 K [18]. These MMM surpassed the Robeson’s upper bound at 5 wt % loading even at increasing temperature, but the separation of CO_2_/N_2_ mixtures with a 12.5 wt % CO_2_ content resulted in a real separation factor much lower than the intrinsic selectivity of the membrane material.

Besides the large number of research and publications devoted to new MMM material combinations for gas separation, there is also a growing literature on the development of systematic approaches to describe gas transport through MMMs [19,20,21]. The MMM performance has been evaluated as a function of the membrane morphology imposed by the filler loading and several models have been compared lately [22,23,24,25]. They all present several limitations such as not being valid but at low filler loadings, a large number of adjustable parameters, or not being able to predict the non-idealities common in MMM morphologies that influence their gas separation performance. The most accurate models reported so far are those proposed by Moore et al. [26] and Li et al. [27], accounting for the void interphase, which describes the compatibility between the zeolite filler and the polymer continuous matrix, and the polymer chain rigidification caused by the effect of the inorganic particles embedded in the polymer matrix, in the first case. The second one distinguishes the transport of fast and slow gas molecules, respectively, and introduces the effect of pore blockage that may become important when the dispersed phase is a porous particle as zeolites are [25]. In fact, partial pore blockage has been recently proven to be the dominant effect when porous zeolites are used as fillers in Matrimid, impeding the increase of permeability with increasing dispersed phase loading [28], in agreement with most studies dealing with low permeability polyimides like Matrimid, polysulfone (PSf), and polyethersulfone (PES). The effect of temperature in the performance of those models is seldom reported [29,30].

Thus, in this work the gas permeation through MMMs prepared from small pore zeolites of different topology and constant Si/Al = 5 in PTMSP is evaluated by modified Maxwell models including the void thickness, chain immobilization and pore-blockage effects, and their variation with temperature.

## 2. Materials and Methods

The MMMs were prepared by a solution-casting method from PTMSP (ABCR, Gelest) previously dissolved in toluene, and CHA, RHO and LTA zeolites of Si/Al = 5 prepared at the Instituto de Tecnología Química (UPV-CSIC) as reported in our previous work [18]. The characteristics of the zeolite fillers used in this work are summarized in Table 1. The membranes were stored in plastic Petri dishes and they were immersed in methanol for a few minutes before gas permeation experiments to remove the effect of aging [31]. The density of the PTMSP pure membranes is 0.75 g/cm^3^.

**Figure 1:**
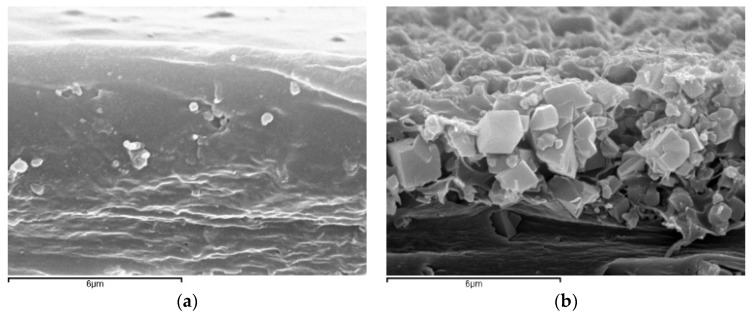

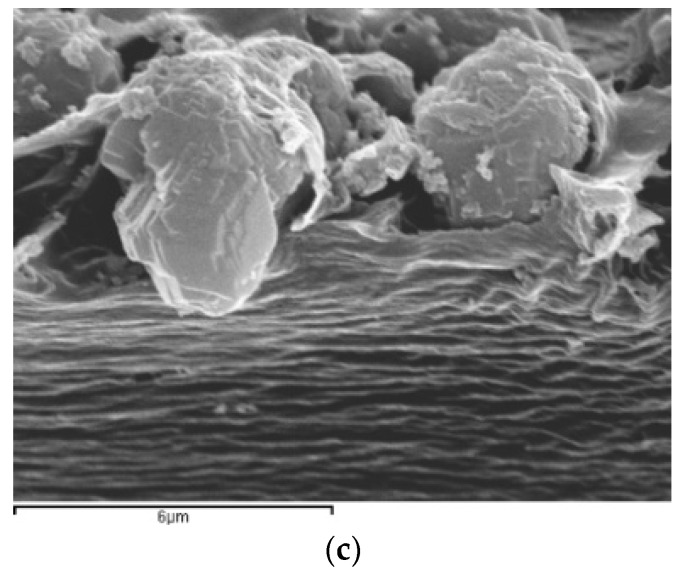
Scanning electron microscope (SEM) images of the detailed contact between LTA (**a**); CHA (**b**); RHO (**c**) and poly(trimethylsilyl-1-propyne) (PTMSP) in 5 wt % loaded mixed matrix membranes (MMMs). Bars correspond to 6 µm.

Figure 1 shows the high magnification scanning electron microscope (SEM) images of 5 wt % CHA, LTA, and RHO/PTMSP MMMs. As reported in a previous work [18], the smaller LTA particles are dispersed throughout the whole membrane thickness, of which a small glimpse can be seen in Figure 1a, while the larger CHA and RHO zeolites form a bottom layer of particles bound together by the polymer, as observed in Figure 1b for a CHA/PTMSP MMM. In the case of RHO, this adhesion is so strong that individual crystals are not easily discerned in Figure 1c. In this work, we want to focus on the compatibility and adhesion between the filler and the polymer, as the main challenge in MMM fabrication [34,35], thus it is important to notice in Figure 1 that even the largest particles at the bottom of the membrane are apparently well adhered with the polymer continuous matrix.

The thickness of every MMM is measured experimentally at 5 points over the membrane surface for each membrane sample using a IP-65 Mitutoyo digital micrometer (Kawasaki, Japan) with a precision of 0.001 mm. The average thickness for all the MMMs tested in this work was 75 ± 14 µm.

The single gas permeation of N_2_ and CO_2_ was measured in that order, using a home-made constant volume set-up described elsewhere [14,18], in the temperature range 298 to 333 K and a feed pressure of 3–4 bar and atmospheric permeate pressure. The average values of the permeabilities and selectivities obtained previously and used in this work are collected in Table A1 in Appendix A.

## 3. Results and Discussion

### 3.1. Comparison of Known Mixed-Matrix Membrane Model Predictions

First, well-known models for predicting MMM permeation (Appendix B) have been compared in terms of the percentage average absolute relative error (AARE) with the permeability of CO_2_ and N_2_ through MMMs, as

(1) $$\mathrm{AARE}\left(\%\right)=\frac{100}{N}\overset{N}{\underset{i=1}{\sum }}\left|\frac{P_{i}^{calc}-P_{i}^{exp}}{P_{i}^{exp}}\right|$$

where *N* is the number of experimental data points [23].

A Maxwell model often represents the ideal case with no defects and no distortion of separation properties. Table 2 summarizes the AARE values obtained with the models most commonly encountered in the literature, averaged for the whole range of temperature studied in our laboratory to allow comparison.

According to Table 2, N_2_ permeability values cannot be predicted by the series, parallel, Maxwell and Higuchi models with acceptable error in all the range of temperature under study. The prediction accuracy of CO_2_ permeability varies as a function of the zeolite topology. Regarding CO_2_ permeability, the series and parallel model approaches fit the 5 wt % CHA/PTMSP MMM performance at 323 K, with a lower average AARE for this membrane. The CO_2_ permeability of LTA/PTMSP MMMs can be described by parallel, Maxwell and Higuchi models in the whole range of operating temperatures and LTA loadings, while the series model only fits the experimental data at low loading. As for the RHO/PTMSP MMM, this is only valid up to 10 wt % RHO loading in the PTMSP matrix. This agrees with the data reported for other MMMs prepared with dispersed fillers of RHO topology [36] where the Maxwell equation only describes the CO_2_ permeability at low loading, as observed for the ZIF-20/Matrimid MMM, being ZIF-20 a zeolite imidazolate framework of RHO topology as well [36]. In the case of our RHO/PTMSP MMMs, all previous models overestimate the experimental permeabilities.

**Figure 2:**
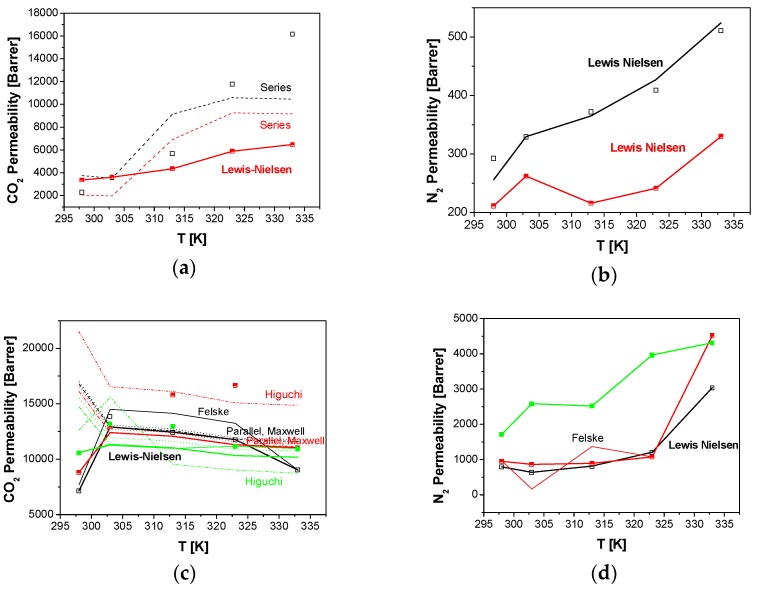

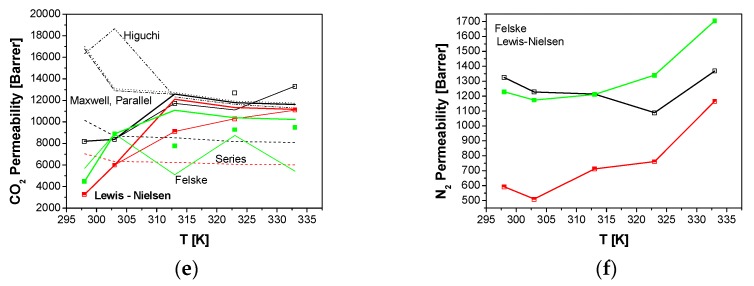
Comparison of CO_2_ (left) and N_2_ permeabilities through CHA (**a**,**b**), LTA (**c**,**d**) and RHO (**e**,**f**)/PTMSP MMMs with the predictions by the series (dashed lines), parallel (dotted lines), original Maxwell (dash-dot), Higuchi (dash dot dot), Felske (thin continuous line) and Lewis–Nielsen (thick continuous line) models, as a function of temperature. Zeolite loading: 5 wt % (black), 10 wt % (red), 20 wt % (green).

Only the model predictions with AARE lower than 20% are represented in Figure 2, for clarification purposes. The original Maxwell equation overestimates the experimental value for the permeability of all gases and membranes, especially for N_2_ permeability. This overestimation is more significant at lower operation temperatures, as reported by Clarizia et al. [14]. In this work, this is true for CHA/PTMSP MMMs with the series model, Figure 2a, and the parallel and Maxwell model for LTA/PTMSP MMMs, Figure 2c. These are simplifications of the general Maxwell equation expressed by Equation (B1) to predict the overall steady-state permeability through an ideal defect-free MMM [26]. Those models provide a simple, quantitative framework to predict the transport properties of MMM when the transport properties of the constituent phases are known, especially at low dispersed phase loading. Only more advanced modifications of this Maxwell equation, such as Felske and Lewis–Nielsen, provide enough accuracy for the description of MMM performance, especially in the case of the slow permeating gas, N_2_, as reflected in Figure 2b,d,f.

### 3.2. Reduced Mobility Modified Maxwell Model

**Figure 3:**
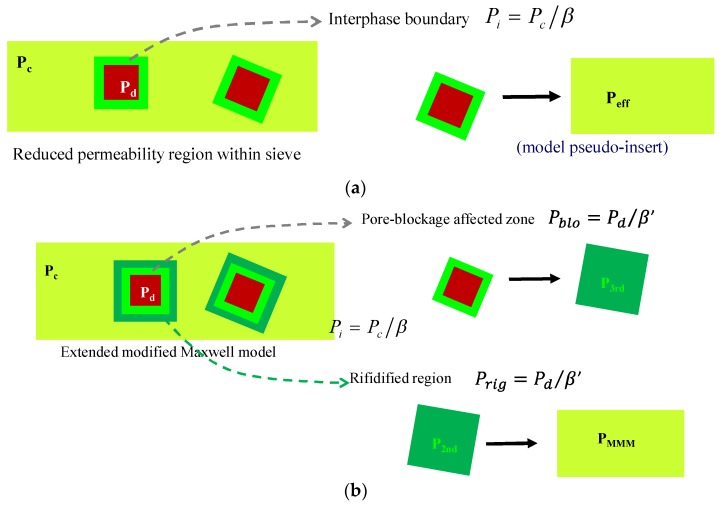
Schemes of the modified Maxwell model proposed by Moore et al. [26] (**a**) and the extended modified Maxwell model proposed by Li et al. [27] (**b**), both adapted for this work.

In order to account for the non-idealities in the membrane morphology accounting for the compatibility that influence the membrane performance [30], polymer chain rigidification and interphase void thickness, the Maxwell model is applied twice to predict the permeability of a pseudo-interphase induced by the interfacial contact between filler and polymer matrix [25], as schematized in Figure 3a.

According to the reduced mobility modified Maxwell model, the effective permeability through the pseudo-insert in Figure 3a, *P*_eff_, is calculated first by

(2) $$P_{\mathrm{eff}}=P_{I}\left[\frac{P_{d}+2P_{I}-2\varphi_{s}\left(P_{c}-P_{d}\right)}{P_{d}+2P_{I}+\varphi_{s}\left(P_{c}-P_{d}\right)}\right]$$

where *ϕ*_d_ is the filler volume fraction in the polymer matrix, *P*_I_ is the permeability through the rigidified continuous matrix, calculated as the ratio between the experimental permeability through a pure PTMSP membrane [18] and an adjustable parameter, *β*, as described in Figure 3a, and *P*_d_ is the permeability through the zeolite. In this work, this value has been taken from literature data on CO_2_ and N_2_ permeation through pure zeolite membranes of similar Si/Al ratio and topology (Table 3) to avoid the usual dispersion on this parameter when calculated from experimental solubility isotherms [23].

In Equation (2), *P*_I_ acts as the permeability of the continuous phase, considering as such the interphase, assuming the bulk of the zeolite as the dispersed phase and the affected zeolite interphase with reduced permeability as the continuous phase [39], as represented in the scheme in Figure 3a. *ϕ*_s_ is the volume fraction of the dispersed sieve phase in combined sieve and interphase, given by

(3) $$\varphi_{s}=\frac{\varphi_{d}}{\varphi_{d}+\varphi_{I}}=\frac{r_{d}^{3}}{{\left(r_{d}+l_{I}\right)}^{3}}$$

where *ϕ*_I_ is the volume fraction of the interface, and *l*_I_ is the thickness of the ‘interface void’. The permeability of the whole MMM is thus estimated by applying the Maxwell equation again, as

(4) $$P_{\mathrm{MMM}}=P_{c}\left[\frac{P_{\mathrm{eff}}+2P_{c}-2\varphi_{s}\left(P_{c}-P_{\mathrm{eff}}\right)}{P_{\mathrm{eff}}+2P_{c}+\varphi_{s}\left(P_{c}-P_{\mathrm{eff}}\right)}\right]$$

As *ϕ*_d_ + *ϕ*_I_ increases to one, the interphases of neighboring dispersed particles overlap and the overall mixed matrix is rigidified. This occurs preferentially as the zeolite particle loading is increased or the interphase void distance is increased, i.e., voids appear because embedding in the polymer chains becomes more difficult.

Equations (2)–(4) predict the overall performance of MMMs taking into account the case morphologies identified by Moore et al. [26], adapted to distinguish the performance of the fast and slow gas in CO_2_/N_2_ separation, and including the influence of temperature. This model is thus based on three adjustable parameters, the interphase thickness, *l*_I_, and the chain immobilization factor, *β*, which depends on the permeating gas molecule [39], whose values are presented in Table 4, Table 5 and Table 6 for the CHA/PTMSP, LTA/PTMSP and RHO/PTMSP MMM, respectively.

As expected, the chain immobilization factor, *β*, is smaller for CO_2_ than N_2_. This confirms that the polymer chain rigidification normally results in a larger resistance to the transport of the gas with larger molecular diameter [27]. The RHO/PTMSP MMM revealed a different trend, although only at 298 K, which may be attributed to the agglomeration of these larger crystal size and smaller pore size particles at the bottom of the MMM. Interestingly, *β*(CO_2_) and *β*(N_2_) of the three types of MMMs converge to similar values upon increasing temperature. This may be attributed to the compensating effects of polymer flexibility and chain rigidification of the polymer matrix, which are accentuated for the larger size of the RHO particles than LTA and CHA. This agrees with the current statement that in gas separation through MMMs there is not only an optimum in zeolite loading but also in operating temperature [40].

The thickness of the interphase between the zeolite and the polymer matrix, *l*_I_ (μm), accounts for the compatibility between the zeolite and polymer phases, as well as the defects or voids due to poor compatibility between zeolites and polymer [25]. In this work, the void thickness decreases with increasing zeolite loading and is independent of the type of gas and temperature. It can also be observed that this parameter *l*_I_ is influenced by the zeolite topology, in the following order: *l*_I_ (LTA/PTMSP) < *l*_I_ (CHA/PTMSP) < *l*_I_ (RHO/PTMSP). This is attributed to the different interaction with the polymer matrix, and the decreasing particle size, in agreement with results obtained for zeolite-APTES/PES MMMs [27]. Those authors obtained as thickness of the rigidified region *l*_i_ = 0.30 µm for a cubic zeolite A (Si/Al = 1) dispersed phase in PES, and values of the chain immobilization factor (*β*) of 3 and 4, for O_2_ and N_2_, respectively. A rigidified thickness of 1.4 µm and chain immobilization factor was reported for ZIF-20/polysulfone MMMs, estimating a *P*_d_ = 45 Barrer, in agreement with pore ZIF membranes of similar pore size and topology [41]. Therefore, the magnitude of the adjustable parameters obtained in this work are in the same order of magnitude.

These parameters allow a prediction of the permeability through these MMMs by this model with an error of up to a global AARE below 6 ± 1%, where the maximum errors lie on 10CHA/PTMSP and 10RHO/PTMSP membranes at 298 K.

### 3.3. Extended Pore-Blockage Reduced Mobility Modified Maxwell Model

Although in this work the channel opening of the zeolites (0.38, 0.41 and 0.36 nm for CHA, LTA and RHO topologies, respectively) lie in the same range as the gas pair molecules to be separated, we have included the analysis of the partial pore blockage effect [25,35] as Li et al. [27] for zeolite A-APTES/PES MMM, adapted in the Scheme shown in Figure 3b. This approach consists in applying the Maxwell equation not just twice, but three times, and requires not just three, but six adjustable parameters, in order to define the dispersed phase volume fraction in the pore-blockage and the rigidified region, as well as the immobilization factor for the pair of gases in both sections.

Firstly, the permeability in the pore-blockage affected zone near the zeolite particle surface as represented in Figure 3b, is calculated by

(5) $$P_{3\mathrm{rd}}=P_{\mathrm{blo}}\left[\frac{P_{d}+2\left(P_{d}/\beta^{\prime }\right)-2\varphi_{3}\left(\left(P_{d}/\beta^{\prime }\right)-P_{d}\right)}{P_{d}+2\left(P_{d}/\beta^{\prime }\right)+\varphi_{3}\left(\left(P_{d}/\beta^{\prime }\right)-P_{d}\right)}\right]$$

Secondly, the *P*_3rd_ permeability calculated by Equation (5) is entered as the new dispersed phase, and the permeability of the rigidified region, *P*_rig_, is taken as the continuous phase, to calculate the new *P*_eff_, *P*_2nd_:

(6) $$P_{2\mathrm{nd}}=P_{\mathrm{rig}}\left[\frac{P_{3\mathrm{rd}}+2\left(P_{c}/\beta \right)-2\varphi_{2}\left(\left(P_{c}/\beta \right)-P_{3\mathrm{rd}}\right)}{P_{3\mathrm{rd}}+2\left(P_{c}/\beta \right)+\varphi_{2}\left(\left(P_{c}/\beta \right)-P_{3\mathrm{rd}}\right)}\right]$$

Thirdly and lastly, the permeability through the bulk of the MMM is calculated using *P*_2nd_ as the new permeability for the dispersed phase, turning the previous equations into

(7) $$P_{\mathrm{MMM}}=P_{c}\left[\frac{P_{2\mathrm{nd}}+2P_{c}-2\left(\varphi_{d}+\varphi_{\mathrm{blo}}+\varphi_{\mathrm{rig}}\right)\left(P_{c}-P_{2\mathrm{nd}}\right)}{P_{2\mathrm{nd}}+2P_{c}+\left(\varphi_{d}+\varphi_{\mathrm{blo}}+\varphi_{\mathrm{rig}}\right)\left(P_{c}-P_{2\mathrm{nd}}\right)}\right]$$

with

(8) $$\varphi_{3}=\frac{\varphi_{d}}{\varphi_{d}+\varphi_{\mathrm{blo}}}$$

and

(9) $$\varphi_{2}=\frac{\varphi_{d}+\varphi_{\mathrm{blo}}}{\varphi_{d}+\varphi_{\mathrm{blo}}+\varphi_{\mathrm{rig}}}$$

Now, the adjustable parameters are *ϕ*_blo_ and *ϕ*_rig_, the calculated volume fraction of the pore-blockage affected region, and the rigidified region, respectively, as well as *β*′ and *β*, whose values depend on the permeating gas, and identify the partial pore blockage affected and rigidified polymer region, respectively, as given in Figure 3b. Note that *β* is similar to the chain immobilization factor introduced by the previous reduced mobility modified Maxwell model, discussed in the previous section.

**Figure 4:**
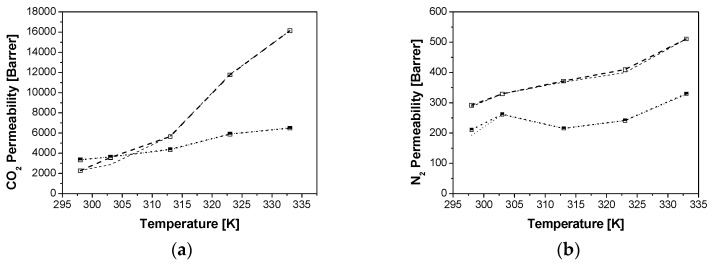
Effect of temperature and zeolite loading on the CO_2_ (**a**) and N_2_ (**b**) permeability through CHA/PTMSP MMMs: Thin lines correspond to the reduced mobility modified Maxwell model and thick lines to the extended modified Maxwell model. Dash, dot and continuous patterns, and void, half-filled and full symbols, refer to 5 wt %, 10 wt % and 20 wt % zeolite loading, respectively.

**Figure 5:**
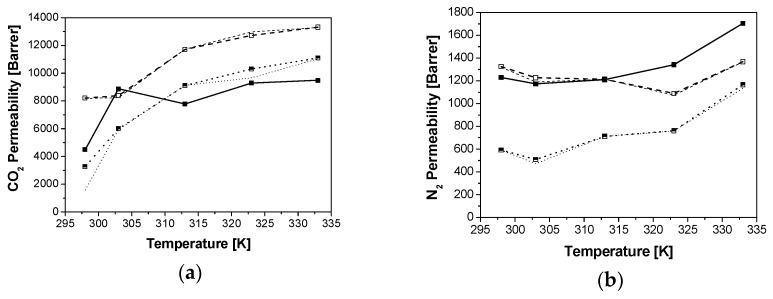
Effect of temperature and zeolite loading on the CO_2_ (**a**) and N_2_ (**b**) permeability through LTA/PTMSP MMMs: Thin lines correspond to the reduced mobility modified model and thick lines to the extended modified Maxwell model. Dash, dot and continuous patterns, and void, half-filled and full symbols, refer to 5 wt %, 10 wt % and 20 wt % zeolite loading, respectively.

**Figure 6:**
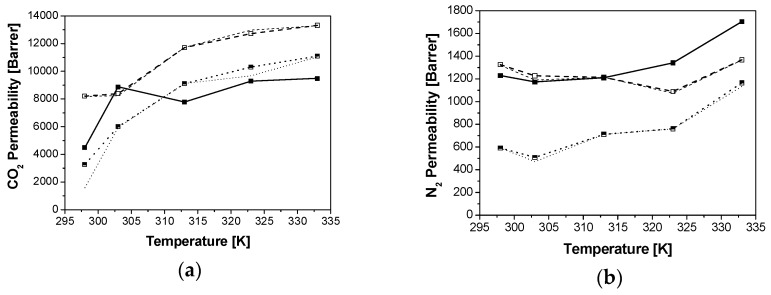
Effect of temperature and zeolite loading on the CO_2_ (**a**) and N_2_ (**b**) permeability through RHO/PTMSP MMMs: Thin lines correspond to the reduced mobility modified model and thick lines to the extended modified Maxwell model. Dash, dot and continuous patterns, and void, half-filled and full symbols, refer to 5 wt %, 10 wt % and 20 wt % zeolite loading, respectively.

Figure 4, Figure 5 and Figure 6 show the comparison of the prediction of CO_2_ and N_2_ permeability using both modified Maxwell models. The experimental results are well described for the Si/Al = 5 zeolites, indicating a good compatibility between intermediate Si/Al zeolites and the glassy PTMSP [14]. The optimized *β* value is higher for N_2_ than CO_2_, for CHA and RHO/PTMSP MMMs. *β*(N_2_) values of 0.92 are obtained for the CHA/PTMSP MMMs, independently of zeolite loading, where as they increase from 0.66 to 1.40 for the RHO/PTMSP MMMs. *β*(CO_2_) gives smaller values than *β*(N_2_), as expected for smaller molecules. *β*(CO_2_) follows similar trends as *β*(N_2_), being constant for CHA and LTA/PTMSP MMMs, at values of 0.3 and 0.2, respectively, and increasing from 0.26 to 0.94 with increasing loading for RHO/PTMSP MMMs. These values are smaller than 1.6, the value recently published for Sigma-1/Matrimid MMMs, considering also the partial pore blockage effect [28]. The values of *β*′(CO_2_) are 0.06 for CHA and RHO/PTMSP MMMs, and below 0.03 for LTA/PTMSP MMMs. The *β*′(N_2_) are 70% higher in the LTA and RHO/PTMSP MMMs, and 30% higher than *β*′(CO_2_) in the case of CHA/PTMSP MMMs. These results reveal that, although the partial pore blockage is low in small–pore zeolites, it is more significant for the smaller pore size zeolite fillers as CHA or RHO, than LTA.

The models describe well the CO_2_ and N_2_ permeability through the Si/Al = 5 zeolite/PTMSP MMMs as a function of zeolite loading, topology and temperature. The CO_2_ permeability increases with temperature while the N_2_ permeability slightly increases for CHA and RHO/PTMSP MMMs, behavior similar to pure zeolite membranes, as reflected by the activation energies derived from the Arrhenius equation in the previous work [18], in agreement with other works in literature [42]. The LTA/PTMSP MMMs show a maximum performance at 10 wt % zeolite loading and 323 K, losing permselectivity at higher loading and temperature. The worst AARE for the prediction of experimental permeabilities through the extended partial pore blockage reduced mobility model is 0.6%, for the 5 wt % CHA/MMM at 313 K, which were in some of the best agreement with the first modified Maxwell model. Partial pore blockage may be affecting permeability even with small-pore zeolite fillers in a glassy polymer matrix [28].

## 4. Conclusions

The experimental CO_2_ and N_2_ permeabilities of Si/Al = 5 small-pore zeolites/PTMSP MMM has been compared with modified Maxwell model predictions as a function of zeolite topology (CHA, LTA, RHO), loading (0–20 wt %) and temperature (298–333 K). Three adjustable parameters accounting for the membrane rigidification, void interphase and partial pore-blockage have been optimized at values lower than reported in literature. They reveal the compatibility between Si/Al = 5 zeolites dispersed in the glassy polymer PTMSP, as well as a small influence of partial pore blockage in the case of the smaller pore size CHA and RHO. The CO_2_ and N_2_ permeabilities through these membranes are predicted with an AARE lower than 0.6% taking into account zeolite loading and topology on non-idealities such as membrane rigidification and sieve pore blockage and their influence on MMM performance. The evolution of this structure-performance relationship with temperature has also been predicted. The implementation of the Arrhenius dependency of the MMM permeability and the prediction studied in this work constitute a step further towards the understanding of the MMM performance in order to develop new membrane materials and module configurations with potential application in CO_2_ separation, which will be addressed in a future work.

## Figures and Tables

**Table 1:** Properties of the zeolite fillers with Si/Al = 5 used in this work.

Filler	Crystal Size (µm)	Density (g/cm^3^)	Pore Size ^1^ (nm)	Structure ^2^
LTA	0.5	1.498 [32]	0.41	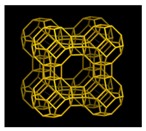
CHA	1.0	2.090	0.38	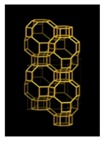
RHO	1.5	1.442 [33]	0.36	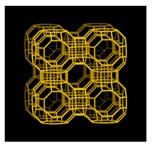

^1^ From [18]. ^2^ The crystallographic structures have been taken from the International Zeolite Database (http://www.iza-structure.org/databases/): View of the planes 100 for LTA and 001 for CHA and RHO, respectively.

**Table 2:** Percentage of average absolute relative error (AARE) for CO_2_ and N_2_ permeation (first and second values in every entry) prediction, highlighting those AARE values lower than 20%.

MMM	Series	Parallel	Maxwell	Higuchi	Felske	Lewis-Nielsen
5CHA/PTMSP	**17.32**/370	108/2026	106/2006	146/2609	118/32.4	24.9/**2.14**
10CHA/PTMSP	24.2/143	102/2966	99.7/2909	96.8/2854	80/936	**10^−4^/10^−5^**
5LTA/PTMSP	20.6/33.3	**11.8**/516	**11.4**/498	26.3/708	**2.54/10^−3^**	**0.46/0.01**
10LTA/PTMSP	40.9/50.0	**14.5**/631	**4.79**/214	**14.6**/560	67.4/**9.04**	**3.98/10^−5^**
20LTA/PTMSP	45.0 /50.0	**7.11** /212	**8.28**/198	**10.4**/194	**3.00**/**10^−4^**	**4.37/10^−5^**
5RHO/PTMSP	**8.62**/126	**12.7**/362	**12.4**/357	**16.7**/395	**0.85/6·10^−4^**	**1.84/0.6·10^−5^**
10RHO/PTMSP	24.0/216	57.0/1030	54.5/1003	49.3/947	**0.03/2·10^−3^**	**4.32/0.02**
20RHO/PTMSP	45.3/52.4	72.2/947	63.8/892	44.2/756	**22.0/5·10^−4^**	**12.3/10^−4^**

**Table 3:** Permeability data of the pure zeolite dispersed phase, *P*_d_, used for the model predictions.

Zeolite Dispersed Phase	*P*_d_(CO_2_) (Barrer)	*P*_d_(N_2_) (Barrer)	T (K)	Reference
CHA (Si/Al = 5) ^1^	88	0.59	293	[37]
CHA (pure silica)	539	55	313	[38]
LTA (Si/Al = 1)	139	0.048	298	[25]
RHO ^2^	623	260	298	[33]

^1^ Si/Al = 5 as the zeolites used in this work. ^2^ The CO_2_ permeabilities reported for ZIF-8 composite values are considered as the Rho here, given the similar sodalite topology.

**Table 4:** Parameters estimated by the reduced mobility modified Maxwell model for the CHA/PTMSP MMMs.

T (K)	5 wt %		10 wt %	
	*l*_I_ (µm) = 1.39		*l*_I_ (µm) = 0.98	
	β (CO_2_)	β (N_2_)	β (CO_2_)	β (N_2_)
298	7.42	61.2	4.90	86.61
303	4.56	53.28	3.48	64.0
313	2.25	42.8	2.87	70.5
323	1.01	31.41	1.97	50.4
333	0.73	20.5	1.00	10.2

**Table 5:** Parameters estimated by the reduced mobility modified Maxwell model for the LTA/PTMSP MMMs.

T (K)	5 wt %		10 wt %		20 wt %	
	*l*_I_ (µm) = 0.60		*l*_I_ (µm) = 0.56 ± 0.08		*l*_I_ (µm) = 0.27	
	β (CO_2_)	β (N_2_)	β (CO_2_)	β (N_2_)	β (CO_2_)	β (N_2_)
298	2.35	21.9	1.83	17.4	1.39	8.82
303	0.93	27.1	1.00	12.0	0.86	5.84
313	1.01	18.9	0.80	11.0	0.85	5.37
323	1.00	10.2	0.72	8.34	0.92	2.72
333	1.29	3.38	1.06	2.49	0.93	2.08

**Table 6:** Parameters estimated by the reduced mobility modified Maxwell model for the RHO/PTMSP MMMs.

T (K)	5 wt %		10 wt %		20 wt %	
	*l*_I_ (µm) = 1.76		*l*_I_ (µm) = 1.23		*l*_I_ (µm) = 0.79	
	β (CO_2_)	β (N_2_)	β (CO_2_)	β (N_2_)	β (CO_2_)	β (N_2_)
298	2.06	0.31	10.62	1.95	3.36	1.46
303	1.57	0.35	2.10	2.98	1.28	1.54
313	1.07	0.30	1.33	1.29	1.43	1.33
323	0.91	0.28	1.17	0.93	1.12	0.93
333	0.87	0.17	1.01	0.45	1.08	0.58

## Appendix A

The experimental permeation data obtained in a previous work [18] are collected in Table A1.

**Table A1:** Experimental data of the different MMMs with increasing order of particle size (LTA, 0.5 µm; CHA, 1 µm; RHO, 1.5 µm).

Filler and Loading [18]	T (K)	P(CO_2_) (Barrer)	P(N_2_) (Barrer)	α(CO_2_/N_2_)
5 wt % LTA	298	7150	794	9
	303	13,881	637	22
	313	12,448	816	15
	323	11,770	1208	10
	333	9026	3044	3
10wt % LTA	298	8813	951	9
	303	12,921	865	15
	313	15,802	892	18
	323	16,648	1078	15
	333	11,029	4520	2.5
20 wt % LTA	298	10,587	1720	6
	303	13,178	2585	5
	313	12,980	2519	5
	323	11,175	3966	3
	333	10,964	4316	2.5
5 wt % CHA	298	2274	292	8
	303	3575	329	11
	313	5651	372	15
	323	11,772	409	29
	333	16,145	511	32
10 wt % CHA	298	3363	211	16
	303	3620	262	14
	313	4351	216	20
	323	5892	241	24
	333	6485	330	20
5 wt % RHO	298	8205	1325	6
	303	8383	1227	7
	313	11,722	1214	10
	323	12,726	1089	12
	333	13,324	1368	10
10 wt % RHO	298	3262	592	6
	303	5996	509	12
	313	9111	712	13
	323	10,304	761	14
	333	11,114	1166	10
20 wt % RHO	298	4479	1229	4
	303	8883	1173	8
	313	7784	1210	6
	323	9293	1341	7
	333	9498	1704	6

## Appendix B

The MMM performance has been evaluated as a function of the membrane morphology imposed by the filler loading using several models that have been compared lately [20,23,24,25]. Equation (A1) was derived by Maxwell for semi-conductors and is widely accepted as an easy tool for a quick estimation of the performance of MMMs from phase-separated blends [3,30]:

(A1) $$P_{\mathrm{mmm}}=P_{c}\left[\frac{P_{d}+2P_{c}-2\varphi_{d}\left(P_{c}-P_{d}\right)}{P_{d}+2P_{c}+\varphi_{d}\left(P_{c}-P_{d}\right)}\right]$$

where *ϕ*_d_ is the dispersed phase volume fraction, calculated from the nominal weight fraction of the zeolite in the MMMs, using the density of the PTMSP polymer and the corresponding zeolite density (Table 1).

The minimum value of effective permeability of a given penetrant in a MMM is given by considering a series mechanism of transport through the dispersed and continuous phases (Equation (A2)):

(A2) $$P_{\mathrm{mmm}}=\frac{P_{c}P_{d}}{\left(1-\varphi_{d}\right)P_{d}+\varphi_{d}P_{c}}$$

and the maximum value is taken when both phases are assumed to contribute in parallel to the flow direction (Equation (A3)):

(A3) $$P_{\mathrm{mmm}}=\varphi_{d}P_{d}+\left(1-\varphi_{d}\right)P_{c}$$

Other important models used for the description of gas permeation in MMMs are the Higuchi, Felske and Lewis–Nielsen, Bruggemann and Pal models [20]. The last two are not presented in this work because they are implicit equations derived from Maxwell and Lewis–Nielsen that have to be solved numerically.

The Higuchi model is applied for a random dispersion of spherical filler particles but lacks mathematical rigor [24]. The main equation for porous zeolite particle fillers is given by:

(A4) $$P_{\mathrm{mmm}}=P_{c}\left[1+\frac{3\varphi_{d}}{\frac{P_{d}+2P_{c}}{P_{d}-P_{c}}-\varphi_{d}-K\left[\frac{\left(1-\varphi_{d}\right)\left(P_{d}-P_{c}\right)}{P_{d}+2P_{c}}\right]}\right]$$

where *K* is an empirical constant containing shape description, with no physical meaning. In this work, it only adjusts the accepted value of 0.78 for 5 wt % CHA, 5–10 wt % LTA5/PTMSP. 10 wt % CHA/PTMSP is adjusted to *K* = 0.999 and for the rest of the membranes *K* varies randomly between 0.0001 and 0.03 at different temperatures.

The Felske model was originally used for the description of the thermal conductivity of composites of core-shell particles (core particle covered with interfacial layer) and also for permeability measurement. It gives almost the same predictions as the modified Maxwell model and it can be reduced to Maxwell’s when the interfacial layer is absent [25]. It is described by Equations (A5)–(A7), as

(A5) $$P_{\mathrm{mmm}}=P_{c}\left[\frac{2\left(1-\varphi_{d}\right)+\left(1+2\varphi_{d}\right)\left(\beta /\gamma \right)}{\left(2+\varphi_{d}\right)+\left(1-\varphi_{d}\right)\left(\beta /\gamma \right)}\right]$$

with

(A6) $$\beta =\frac{\left(2+\delta^{3}\right)P_{d}-2\left(1-\delta^{3}\right)P_{I}}{P_{c}}=\left(2+\delta^{3}\right)\frac{P_{d}}{P_{c}}-2\left(1-\delta^{3}\right)\frac{P_{I}}{P_{c}}$$

and

(A7) $$\gamma =1+2\delta^{3}-\left(1-\delta^{3}\right)\frac{P_{d}}{P_{c}}$$

where *δ* = *r*_I_/*r*_d_. This model also needs three adjustable parameters, as in the reduced mobility modified Maxwell model.

The Lewis–Nielsen model was originally proposed for describing an elastic modulus of particulate composites, and the following equation can be used to predict the effective permeability in MMMs:

(A8) $$P_{\mathrm{mmm}}=P_{c}\left[\frac{1+2\varphi_{d}\left(\alpha -1\right)/\left(\alpha +2\right)}{1-\psi \varphi_{d}\left(\alpha -1\right)/\left(\alpha +2\right)}\right]$$

where

(A9) $$\psi =1+\left(\frac{1-\varphi_{m}}{\varphi_{m}}\right)$$

This model might represent a correct definition of the permeability over the range of 0 < *ϕ*_d_ < *ϕ*_m_. The solution diverges when *ϕ*_d_ = *ϕ*_m_ and it should be noted that when *ϕ*_m_ → 1, the Lewis–Nielsen model reduces to the Maxwell equation (Equation (A1)).
